# Reversible Axonal Dystrophy by Calcium Modulation in Frataxin-Deficient Sensory Neurons of YG8R Mice

**DOI:** 10.3389/fnmol.2017.00264

**Published:** 2017-08-30

**Authors:** Belén Mollá, Diana C. Muñoz-Lasso, Fátima Riveiro, Arantxa Bolinches-Amorós, Federico V. Pallardó, Angel Fernandez-Vilata, María de la Iglesia-Vaya, Francesc Palau, Pilar Gonzalez-Cabo

**Affiliations:** ^1^CIBER de Enfermedades Raras (CIBERER) Valencia, Spain; ^2^Instituto de Biomedicina de Valencia (IBV), CSIC Valencia, Spain; ^3^Department of Physiology, Faculty of Medicine and Dentistry, University of Valencia-Instituto de Investigación Sanitaria (INCLIVA) Valencia, Spain; ^4^Associated Unit for Rare Diseases INCLIVA-CIPF Valencia, Spain; ^5^VEDAS Corporación de Investigación e Innovación, VEDASCII Medellín, Colombia; ^6^Fundacion Publica Galega de Medicina Xenomica (FPGMX)-SERGAS, Grupo de Medicina Xenomica, Hospital Clínico Universitario Santiago de Compostela, Spain; ^7^Cell Therapy Program, Prince Felipe Research Centre (CIPF) Valencia, Spain; ^8^Regional Ministry of Health in Valencia, Hospital Sagunto (CEIB-CSUSP) Valencia, Spain; ^9^Brain Connectivity Laboratory, Joint Unit FISABIO & Prince Felipe Research Centre (CIPF) Valencia, Spain; ^10^CIBER de Salud Mental (CIBERSAM) Valencia, Spain; ^11^Department of Genetic and Molecular Medicine, Institut de Recerca Sant Joan de Déu, Hospital Sant Joan de Déu Barcelona, Spain; ^12^Department of Pediatrics, University of Barcelona School of Medicine Barcelona, Spain

**Keywords:** rare disease, Friedreich’s ataxia, mitochondria, calcium, neurodegeneration, axonal spheroids

## Abstract

Friedreich’s ataxia (FRDA) is a peripheral neuropathy involving a loss of proprioceptive sensory neurons. Studies of biopsies from patients suggest that axonal dysfunction precedes the death of proprioceptive neurons in a dying-back process. We observed that the deficiency of frataxin in sensory neurons of dorsal root ganglia (DRG) of the YG8R mouse model causes the formation of axonal spheroids which retain dysfunctional mitochondria, shows alterations in the cytoskeleton and it produces impairment of axonal transport and autophagic flux. The homogenous distribution of axonal spheroids along the neurites supports the existence of continues focal damages. This lead us to propose for FRDA a model of distal axonopathy based on axonal focal damages. In addition, we observed the involvement of oxidative stress and dyshomeostasis of calcium in axonal spheroid formation generating axonal injury as a primary cause of pathophysiology. Axonal spheroids may be a consequence of calcium imbalance, thus we propose the quenching or removal extracellular Ca^2+^ to prevent spheroids formation. In our neuronal model, treatments with BAPTA and *o*-phenanthroline reverted the axonal dystrophy and the mitochondrial dysmorphic parameters. These results support the hypothesis that axonal pathology is reversible in FRDA by pharmacological manipulation of intracellular Ca^2+^ with Ca^2+^ chelators or metalloprotease inhibitors, preventing Ca^2+^-mediated axonal injury. Thus, the modulation of Ca^2+^ levels may be a relevant therapeutic target to develop early axonal protection and prevent dying-back neurodegeneration.

## Introduction

Friedreich’s ataxia (FRDA) is a neurodegenerative disease characterized by progressive limb and gait ataxia associated with hypertrophic cardiomyopathy and diabetes mellitus. Early pathological changes appear in the dorsal root ganglia (DRG) and sensory peripheral nerves leading to gait ataxia. Peripheral neuropathy consists of hypomyelination and axonal loss of large myelinated sensory neurons of DRG with slowly dying-back degeneration (Morral et al., 2010; Koeppen, 2013). Throughout the disease course, neuronal destruction becomes manifest by a loss of cell body sensory neurons, mainly the proprioceptive neurons (Koeppen and Mazurkiewicz, 2013). The late destruction of the dentate nucleus and degeneration of the corticospinal tracts in the CNS are secondary to the DRG lesion (Koeppen et al., 2011; Koeppen and Mazurkiewicz, 2013).

Mutations in the frataxin gene (*FXN*) gene are responsible for the disease. In 98% of patients this disorder is caused by the homozygous GAA-triplet repeat expansion (Campuzano et al., 1997) in the *FXN* first intron associated with epigenetic changes (Festenstein, 2006) affects transcription and reduce the amount of frataxin in all tissues. Only 2% of FRDA alleles carry a point mutation or deletion in sequence. Frataxin is a small protein of 23 kDa which is associated with the mitochondrial inner membrane (Campuzano et al., 1997).

Frataxin participates in different physiological functions including metabolism of mitochondrial iron, biogenesis of iron-sulfur clusters (ISC) and heme domain, response to oxidative stress, mitochondrial biogenesis and regulation of cellular Ca^2+^ homeostasis. In biopsies of FRDA patients, frataxin reduction has been associated with iron deposits (Lamarche et al., 1980), mitochondrial dysfunction (Rötig et al., 1997) and ROS production (Emond et al., 2000; Schulz et al., 2000; Bradley et al., 2004). In this way, functional studies have determined that frataxin is a metallochaperone required for the intracellular iron homeostasis (Adamec et al., 2000), ISC and heme biogenesis (Gerber et al., 2003) and aconitase activation (Bulteau et al., 2004). In addition, frataxin interacts physically with components of the ISC assembly complex (Stemmler et al., 2010) and complex II of the electron transport chain (González-Cabo et al., 2005), directly linking frataxin with mitochondrial structural organization, mitochondrial function and energetic production within the cell (Lodi et al., 1999; Ristow et al., 2000).

Frataxin deficiency in yeast, *Caenorhabditis elegans*, *Drosophila melanogaster* or mouse models have confirmed mitochondrial dysfunction with iron accumulation and hypersensitivity to oxidative stress (Babcock et al., 1997; Al-Mahdawi et al., 2006; Vázquez-Manrique et al., 2006; Llorens et al., 2007). Mitochondrial failure promotes mitochondrial biogenesis in FRDA fibroblasts (García-Giménez et al., 2011), and increases the autophagy in SH-SY5Y human neuroblastoma cell model based on frataxin silencing (Bolinches-Amorós et al., 2014) and in conditional knock-out mouse (Simon et al., 2004). Cellular calcium homeostasis is intimately dependent on functional mitochondria. Therefore, frataxin overexpression in 3T3-L1 adipocytes increased mitochondrial bioenergetics and mitochondrial buffer calcium capacity (Ristow et al., 2000). In contrast, the stable frataxin silencing in SH-SY5Y human neuroblastoma cells induced mitochondrial dysfunction that was associated with decreased mitochondrial buffer calcium capacity (Bolinches-Amorós et al., 2014). Defects in calcium and ATP levels have been related to a failure of retrograde axonal transport and an abnormal distribution of distal mitochondria, contributing to dying-back neuropathy in a *Drosophila* larvae model with reduced frataxin expression (Shidara and Hollenbeck, 2010).

The frataxin deletion in mice causes embryonic lethality (Cossée et al., 2000), but it could be rescued by human frataxin (Pook et al., 2001). The YG8R mouse model is a frataxin knockout mice rescued by a transgene (YG8) that contains the entire FRDA locus from a FRDA patient with the prevalent mutation responsible of the disease, a pathological GAA expansion in intron 1 (Al-Mahdawi et al., 2006). This dynamic mutation is responsible for an improper transcription of human frataxin gene and decrease frataxin protein amounts to pathological level. Therefore, the mouse model YG8R exhibits progressive FRDA-like pathology (Al-Mahdawi et al., 2006). Histological and biochemical studies in neuronal tissues showed alterations in peripheral structures, while the spinal cord and brain were normal. YG8R mice show decreased aconitase activity, increased oxidative stress, motor coordination defects and loss of sensory neurons in DRG and their corresponding axons in nerve roots (Al-Mahdawi et al., 2006; Mollá et al., 2016). Morphological observations in YG8R showed a myelin-sheath decompaction incrementing the adaxonal space and demyelination of axons. All these pathological processes could develop before axon degeneration and contribute to their disappearance (Mollá et al., 2016). The YG8R mouse reproduces the observations on the post-mortem sural nerves of FRDA patients where hypomyelination is present due to impaired interaction of axons and Schwann cells (Morral et al., 2010). Damage in nervous tissues begins in the axons with defects in Schwann cells that lead to the disappearance of neuronal body cells.

It is known that a lack of frataxin causes mitochondrial dysfunction and its consequences on the nervous system are responsible for the neural pathophysiology of the disease. However, the exact role of frataxin and the molecular mechanisms that underlie the dying-back neurodegeneration in FRDA remain unknown. Therefore, we have studied the axonal degeneration process on DRG sensory neurons of YG8R mice. We show that chronic frataxin deficiency induces the failure of mitochondria involving oxidative stress and improper calcium handling, which are responsible for axonal dystrophy and the impairment of axonal transport and autophagic flux. We confirm that Ca^2+^ modulation reverts dysmorphic mitochondria and axonal dystrophy. Thus, pharmacological manipulation of axonal degeneration, through to restored Ca^2+^ homeostasis, may be an early therapeutic target in FRDA to prevent dying-back degeneration and subsequent neuronal death.

## Materials and Methods

### Animals and Dorsal Root Ganglia (DRG) Primary Culture

YG8R mice were purchased from The Jackson Laboratory Repository (stock no. 008398. Maine, USA). The YG8R mouse is a knock-out with both mouse frataxin alleles deleted *(Fxn)* and hemizygous for the transgene “YG8” that contains two tandem copies of the human *FXN* gene with 90 and 190 GAA trinucleotide sequence repeats. The GAA expansions promote a low frataxin expression level but enough to rescue the embryonic lethality in knockout mice (Cossée et al., 2000; Al-Mahdawi et al., 2006). For this study, we used the crossing system and genotyping as previously described (Mollá et al., 2016). We used both hemizygous mice, containing one allele of the transgene, YG8R and homozygous mice, containing two alleles of the transgene, YG8YG8R and C57BL/6J as control mice (control). Animals were group-housed under standard housing conditions with a 12 h light–dark cycle, and food and water *ad libitum*. All mouse experiments were approved by the local Animal Ethics Review Committee of the Consejo Superior de Investigaciones Científicas (CSIC) and the Centro de Investigación Príncipe Felipe (CIPF).

Adult mice were sacrificed by cervical dislocation between 22 and 24 months old and approximately 50 DRGs per animal were dissected and collected in L15 media. Ganglia were incubated at 37°C with 2 mg/ml collagenase (Worthington Biochemical Corporation, Lakewood, NJ, USA) followed by 0.05% trypsin (Sigma-Aldrich, Madrid, Spain). Approximately 50,000–80,000 neurons per animal were obtained in 1 ml of defined medium, consisting of Ham’s F12 supplemented with 2 mM glutamine, 0.35% albumax (Invitrogen™), 60 ng/ml progesterone, 16 μg/ml putrescine, 400 ng/ml L-thyroxine, 38 ng/ml sodium selenite, 340 ng/ml tri-iodo-thyronine, 60 μg/ml penicillin and 100 μg/ml streptomycin supplemented with NGF, BDNF and NT3 at 10 ng/ml and 5 μM Ara-C. Then, 2000 neurons per well were plated in 24-well plates (Sarstedt AG and Co, Nümbrecht, Germany) with 12 mm-coverslips that had been previously coated with poly-ornithine (0.5 mg/ml, overnight) and laminin (20 μg/ml, 4 h). Isolated neurons were incubated at 37°C in a humidified incubator under 5% CO_2_ (Davies et al., 1993).

### Immunocytochemistry

To monitor mitochondria, DRG neurons were incubated with 200 nM Mitotracker deep red (Molecular Probes™) for 15 min at 37°C and rinsed with defined medium 15 min at 37°C. Later, neurons were fixed for 20 min with 4% PFA and 120 mM sucrose in PB 0.1 M, rinsed three times with PB 0.1 M, and blocked in 10% FBS and 0.2% Triton X-100 in PB 0.1 M for 30 min at room temperature. Neurons were then incubated overnight at 4°C with antibodies against β-tubulin III (1:1000, Sigma-Aldrich), peripherin (1:2000, Millipore), β-APP or beta-amyloid precursor protein (1:500, Abcam), synaptophysin (1:250, Sigma-Aldrich), p62 or sequestosome 1 (SQSTM1; 1:100, Abcam), and LAMP2 or Lysosome-associated membrane protein 2 (1:500, DSHB). After three washes with PB 0.1 M, neurons were incubated with appropriate secondary antibodies Alexa Fluor 488 or 633 (Molecular Probes™) for 1 h at room temperature. Following three washes with PB 0.1 M, the nuclei of neurons were stained with DAPI for 15 min at room temperature and then coated with Fluoromount G (Southern Biotech Assoc. Inc, Birmingham, AL, USA). Images were acquired using a Leica TCS SP8 laser-scanning confocal microscope. Confocal imaging settings were kept constant between genotypes and experimental replicates. For confocal z-axis stacks, series of stack images (1024 × 1024) separated by 0.22 μm along the z-axis were acquired to visualize neuronal structures. 2D reconstructions of the stacks were performed with ImageJ (NIH, Bethesda, MD, USA).

### Electron Microscopy

Neurons were seeded at 500 cells/cm^2^ in Permanox chamber (Nalge Nunc International, Naperville, IL, USA) coated with poly-ornithine (0.5 mg/ml, overnight) and laminin (20 μg/ml, 4 h). DRG primary cultures at 5 days *in vitro* (DIV) were fixed in 2.5% glutaraldehyde for 5 min at 37°C and 1 h at 4°C. Next, steps were performed as previously described (Bolinches-Amorós et al., 2014). Photomicrographs were obtained under a transmission electron microscope FEI Tecnai G2 Spirit (FEI Europe) using a digital camera Morada (Olympus Soft Image Solutions GmbH).

### Measurement of Reactive Oxygen Species (ROS)

DRG primary cultures at 5 DIV were incubated for 30 min at 37°C with 3 μM MitoSOX™ Red (Molecular Probes™). MitoSOX™ is a cell-permeable probe that accumulates in the mitochondria and fluoresces following oxidation by superoxide. As a positive control for pro-oxidative or mitochondrial depolarization, C57BL/6J DRG neurons were treated with 500 μM H_2_O_2_ or 4 μM CCCP + 1 μM oligomycin for 30 min prior to and during 30 min incubation with MitoSOX™. The coverslips were fixed for 10 min with 4% PFA and sucrose 120 mM in PB 0.1 M at room temperature and then blocked for 1 h at room temperature. Cultured neurons were identified by immunofluorescence using anti-β-tubulin III and counterstained with DAPI for nuclei visualization. The microscope slides were digitized with a Hamamatsu camera (Tokyo, Japan) connected to a Leica DM RXA2 microscope (Nussloch, Germany). Microscopy imaging settings were kept constant between genotypes and experimental replicates. To quantify ROS production, fluorescence intensity relative to area was measured in neuronal somas with ImageJ. At least 236 neurons were analyzed in three or more independent experiments for each genotype.

### Measurement of Mitochondrial Membrane Potential (Δψ_m_)

Cultured DRG neurons at 5 DIV were incubated at 37°C for 30 min with JC1 dye (3 μM; Molecular Probes™). In healthy neurons, JC1 is taken up by the mitochondria and when a critical concentration is reached, red fluorescent J-aggregates are formed. Loss of Δψ_m_ causes the dye to diffuse into the cytoplasm where it exists in a green fluorescent monomeric form. As a positive control for mitochondrial depolarization, C57BL/6J DRG neurons were treated before with 4 μM CCCP + 1 μM oligomycin for 10 min and during to 30 min incubation with JC1-dye. The coverslips were fixed briefly for 10 min with 0.5% PFA and sucrose 120 mM in PB 0.1 M at room temperature and blocked for 1 h at room temperature (Liu et al., 2013). Cultures were immunodetected with anti-β-tubulin III to identify neurons and counterstained with DAPI for nuclei visualization. The emission of JC-1 monomers and aggregates was detected through 530–550 nm and 585–650 nm emission wavelength ranges respectively, by confocal microscope using a Leica TCS SP8 laser-scanner. Confocal imaging settings were kept constant between genotypes and experimental replicates. 2D reconstructions of the stacks were performed with ImageJ (NIH, Bethesda, MD, USA). To quantify Δψ_m_, fluorescence intensity relative to area was measured in neuronal somas with ImageJ and the fluorescence ratio 590 nm/530 nm was expressed. At least 87 neurons were analyzed in three or more independent experiments for each genotype.

### Measurement of Intracellular Ca^2+^ Concentration [Ca^2+^]_cyt_

Cytosolic calcium imaging with Fura-2 AM (Molecular Probes™) was performed in live DRG neurons cultured at 5 DIV. Neurons were incubated with 5 μM Fura-2 and pluronic acid 0.06% (Sigma-Aldrich) as previously described (Bolinches-Amorós et al., 2014). Neurons were clearly identified by morphological criteria and changes in fluorescence (emission 510 nm) ratio of Ca^2+^-free (380 nm) to Ca^2+^-bound probe (340 nm) were recorded through a 40× fluo oil-immersion objective. Endoplasmic reticulum (ER) calcium storage was depleted by the addition of 150 μM 2,5-di-(ter-butyl)-1,4-benzohydroquinone (tBuBHQ, Alomone Labs) in Ca^2+^-free HCSS medium and later store-operated calcium entry (SOCE) was induced by the addition of 6 mM CaCl_2._ C57BL/6J DRG neurons were pretreated with 4 μM CCCP + 1 μM oligomycin for 10 min as a control of depolarized mitochondria. Fluorescence ratio was analyzed using Leica Metafluor (Universal Imaging). Microscopy imaging settings were kept constant between genotypes and experimental replicates. [Ca^2+^]_cyt_ was calculated from ratio fluorescence Fura-2 measurements following an intracellular calibration procedure according to the Grynkiewicz equation (Grynkiewicz et al., 1985). At least 31 neurons were analyzed in three or more independent experiments for each genotype.

### Measurement of Calpain Activity

Calpain protease activity imaging was performed with CMAC, t-BOC-Leu-Met (Molecular Probes™) in live DRG neurons cultured at 3 DIV. Neurons were incubated in a sequential two-step process with 200 nM of Mitotracker deep red for 15 min at 37°C and 10 μM CMAC, t-BOC-Leu-Met for 15 min at 37°C. Fluorescence (emission 430 nm), corresponding to cleaved t-BOC produced by calpain activity, was monitored in live neuronal imaging using a 40× objective on a Leica TCS SP8 laser-scanning confocal microscope. Confocal imaging settings were kept constant between genotypes and experimental replicates. 2D reconstructions of the stacks were performed with ImageJ (NIH, Bethesda, MD, USA). Cultured neurons were identified by morphological criteria and fluorescence intensity relative to area was measured in neuronal somas with ImageJ. At least 46 neurons were analyzed in three or more independent experiments for each treatment and genotype. For experiments using [Ca^2+^]_cyt_ modulators, DRG cultures at 2 DIV were treated for 24 h with: (i) extracellular calcium chelator, 1 mM EGTA (Sigma-Aldrich); (ii) intracellular calcium chelator, 20 μM BAPTA-AM (Molecular Probes™); (iii) inhibitor of metal-ion-dependent enzymes, 100 μM *o*-phenanthroline (Sigma-Aldrich); and (iv) two-fold increase physiological extracellular calcium, 3.6 mM CaCl_2_.

### Mitochondrial Network Analysis of DRG Neurons in Culture

To performed mitochondrial morphology analysis, DRG neuron mitochondria were labeled with Mitotracker as described above, neurons were immunodetected with anti-β-tubulin III and nuclei were stained with DAPI. C57BL/6J DRG neurons treated previously with 4 μM CCCP + 1 μM oligomycin or 5 μM Br-A23187 for 30 min plus incubation with Mitotracker were used as a control of depolarized mitochondria or intra-axonal overload calcium, respectively. Fluorescence was acquired using a Leica TCS SP8 laser-scanning confocal microscope. Confocal imaging settings were kept constant between genotypes and experimental replicates. 2D reconstructions of the stacks were performed with ImageJ (NIH, Bethesda, MD, USA). Morphometric mitochondrial analysis was performed using mito-morphology macro of ImageJ, created by Dagda et al. (2009) and customized by the Brain Connectivity Laboratory (FISABIO/CIPF), headed by MI-V. The macro will draw individual outlines for each mitochondrion and calculate mitochondria number, circularity, perimeter (μm), area (μm^2^) and % of neuronal area occupied by mitochondria. Index of elongation (inverse of circularity) is sensitive for fragmented to normal-shaped mitochondria and is validated as mitochondrial fission parameter. The index of mitochondrial interconnectivity (area/perimeter ratio) is sensitive for normal to highly interconnected mitochondria that are visualized as a single interconnected mass of reticular network and is validated as mitochondrial fusion parameter. The index of swollen mitochondria (area/perimeter normalized to the minor axis of mitochondria or circularity) is sensitive for swelling mitochondria that attain a large area and may be confounded with interconnected mitochondria; it is validated as accounting for conditions that can induce mitochondrial swelling. For experiments using [Ca^2+^]_cyt_ modulators, DRG cultures were treated for 4 DIV with: (i) 1 mM EGTA (Sigma-Aldrich); (ii) 20 μM BAPTA-AM (Molecular Probes™); (iii) 100 μM *o*-phenanthroline; and (iv) 3.6 mM CaCl_2_.

### Immunodetection of Protein Expression by Western Blot

We studied protein expression in DRG tissue by western blot. Protein extraction, electrophoresis, transference and blocking were performed as previously described (Mollá et al., 2016). Membranes were incubated in blocking buffer overnight at 4°C with primary antibodies against human FXN (1:500, Immunological Sciences), actin (1:1000, Sigma-Aldrich), p62 (1:500, Abcam) and mono- and poly-ubiquitinylated conjugates-biotin conjugate (1:1000, ENZO). The blots were incubated for 1 h with the appropriate secondary anti-rabbit or anti-mouse conjugated with horseradish peroxidase (HRP)-linked (1:5000, GE). For biotin signal amplification, Pierce™ high sensitivity NeutrAvidin™-HRP (1:20,000, Thermo Fischer Scientific) was used. Blots were developed using the ECL Prime Western Blotting Detection Reagents as specified by the manufacturer (GE HealthcareBio-Sciences). Chemiluminescent signals were assessed with X-ray film and processor (Kodak, Rochester, NY, USA). Densitometry was measured using ImageJ software (NIH, Bethesda, MD, USA).

### Statistical Analysis

GraphPad. PRISM^®^ 5.00.288 software (GraphPad Software Inc., La Jolla, CA, USA) was used to generate the graphs and statistical analysis. Experiments were performed at least in three animals per genotype. The normal distribution of each sample of data was determined with Shapiro-Wilk normality test. To determine significant values between different experimental groups, the mean data were compared using one-way analysis of variance (ANOVA), followed by a Bonferroni *post hoc* test. Significant *p-values:* *p < 0.05; **p < 0.01; ***p < 0.001 were considered.

## Results

### Frataxin Deficiency in DRG Culture Leads to Axonal Spheroids Formation and Alterations in Axonal Transport

**Figure 1:**
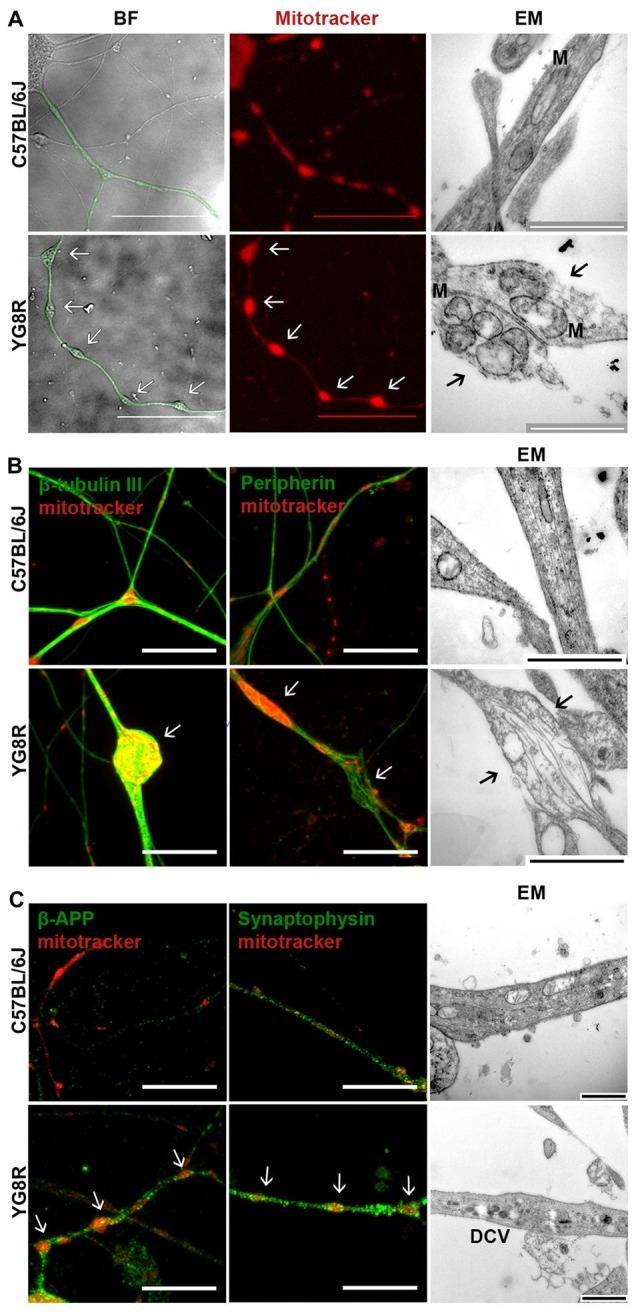
Frataxin-deficient sensory neurons develop axonal spheroids where mitochondrial, cytoskeleton and transport vesicle proteins are retained.** (A)** BF images (left panel) show the neuritic network marked in green. The control lists healthy axonal network while axonal spheroids are developed in YG8R neurons. The mitochondrial labeling with Mitotracker in red (middle panel) shows a proper distribution of mitochondria in control neurons; in contrast, abnormal mitochondrial distribution is observed in the YG8R neurons. Electron microscopy images (EM, right panel) confirm the retention of mitochondria inside the axonal spheroids in frataxin-deficient sensory neurons. The arrows [↑] indicate axonal spheroids with retained mitochondria. M: mitochondria. Scale bar, 50 μm (BF and fluorescence microscopy) and 1 μm (EM). **(B)** C57BL/6J control neurons did not show swollen areas nor axonal spheroids in culture. YG8R neurons showed axonal deformities (arrow) contained accumulations of β-tubulin III microtubules in axonal spheroids (left panel, green) and disorientated peripherin neurofilaments in axonal swellings (middle panel, green). Mitochondria were labeled with Mitotracker (red). Disorganized cytoskeleton in axonal swellings was found in ultrastructure image without fragmentation (arrow, right panel). Scale bar, 10 μm (BF and fluorescence microscopy) and 1 μm (EM). **(C)** Axonal swellings (arrow) developing in YG8R neurons were frequently associated with β-APP and synaptophysin immunoreactivity (left and middle panel, green) demonstrating the accumulation of transport vesicles in swollen axons. Mitochondria were labeled with Mitotracker (red). In the right panel, the high presence of DCVs confirms the impairment of axonal transport. Large DCVs (≈ 100 nm) transport neuroactive peptides and co-transmitters while small DCVs (≈ 80 nm) contain components of the presynaptic active zone. Scale bar, 10 μm (fluorescence microscopy) and 1 μm (EM). DCV, dense core vesicles. Magnification, 63× oil immersion (fluorecence microscopy) and 43,000× amplification (EM, electron microscopy).

The studies were performed in 24-month-old YG8R, YG8YG8R and C57BL/6J mice, since we have observed histopathological changes in very old animals (Mollá et al., 2016). In neural tissue, the expression level of human frataxin was proportional to the number of copies of the transgene (Supplementary Figure S1). Primary cultures of DRG were labeled with Mitotracker at 5 DIV and the frataxin-deficient neurons exhibited axonal swelling and spheroid formation that retained a great amount of mitochondria, in a completely different pattern than observed in control neurons (Figure 1A). Electron microscopy showed that the mitochondria retained in the axonal spheroids in YG8R were more rounded and swollen than in the control (Figure 1A, right panel).

The effect of spheroids formation and mitochondrial retention in frataxin-deficient neurons was a disorganization of the axonal cytoskeleton (Figure 1B). By immunofluorescence, we have frequently observed an accumulation of microtubules in axonal spheroids detected with β-tubulin III and even disorganized neurofilaments of peripherin in axonal swellings (Figure 1B).

The cytoskeleton structure is essential for a proper axonal transport. Thus, neurofilament and microtubules alterations suggest an impairment of transport along axons. To investigate this point, we tested the accumulation of β-APP and synaptophysin, which are vesicle transport proteins and markers of transport disruption (Adalbert et al., 2009). The detection of β-APP-positive spheroids and synaptophysin along the axons suggest a disruption of axonal transport (Figure 1C). Moreover, the presence of dense-core vesicles (DCVs) in electron microscopy images supports this idea (Figure 1C).

**Figure 2:**
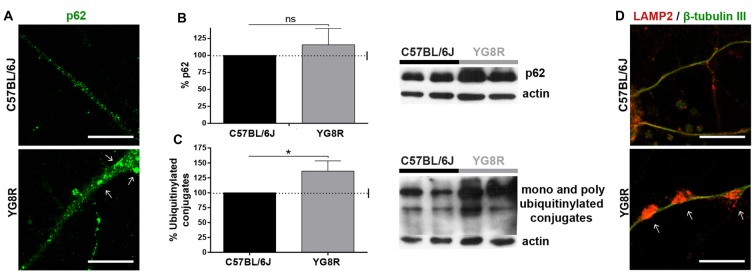
Accumulation of autophagic intermediates in frataxin-deficient neurons. **(A)** Accumulation of the protein p62 (green) in cultured YG8R neurons by immunofluorescence. **(B,C)** Quantitative western blot in YG8R dorsal root ganglia (DRG) tissue to measure the accumulation of p62 **(B)** and ubiquitinylated conjugates **(C)** compared with the control. Values were normalized to the actin control. Final values were expressed as percentage value of the control. Mean ± SEM (*n* = 4 mice of each genotype, assayed in duplicate). T student and Bonferroni *post hoc* test were used to analyze the significance. Significant *p*-values: *p < 0.05 were considered. ns: not significant. **(D)** Immunolabeled lysosomes against LAMP2 protein (red) are retained in axonal spheroids (arrow), β-tubulin III (green). Magnification, 63× oil immersion. Scale bar, 10 μm.

Autophagy in neurons is highly dependent on axonal transport. To know about autophagic flux in frataxin-deficient neurons, we measured autophagic intermediates. In a primary culture of YG8R neurons, we observed an increase of p62 fluorescence (Figure 2A), indicating high levels of ubiquitinylated proteins or damaged mitochondria marked for recycling. The quantification of p62 and ubiquitination in DRG tissue by western blot (Figures 2B,C) showed a significant increase of ubiquitinylated proteins (*p* < 0.05). This accumulation of autophagic intermediates suggests the impairment of autophagic flux that was supported by a huge retention of lysosomes labeled with LAMP2 in axonal spheroids (Figure 2D).

The accumulation of mitochondria, LAMP2, β-APP and synaptophysin in axonal swellings suggests the failed axonal transport of different cargoes, including lysosomes, synaptic vesicles (SV) and DCVs.

### Frataxin Deficiency Causes Mitochondrial Depolarization and Oxidative Stress

**Figure 3:**
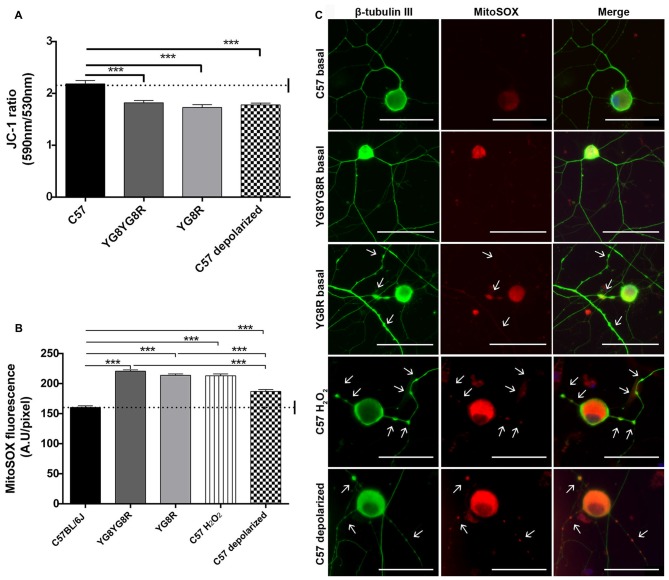
Detection of Δψ_m_ and superoxide anion production in sensory neurons of the Friedreich’s ataxia (FRDA) mouse model. **(A)** Quantitative analysis of JC-1 fluorescence intensity (red/green ratio) detected by confocal microscopy in cultured sensory neurons of control, YG8YG8R, YG8R and control treated with CCCP-oligomycin (C57 depolarized; 182, 182, 129 and 87 total neurons measured respectively from no less than three experiments). We observed mitochondrial depolarization in frataxin gene (FXN)-deficient and depolarized control neurons. **(B)** Quantitative analysis of MitoSOX™ red fluorescence intensity detected in primary cultures of DRG. The fluorescence intensity is presented relative to neuronal soma area (pixel) in YG8R, YG8YG8R, control and control treated with H_2_O_2_ (C57 H_2_O_2_) and CCCP-oligomycin (C57 depolarized) neurons (712, 256, 236, 365 and 287 total neurons measured, respectively, from no less than three experiments). We observed ROS production associated with mitochondrial depolarization in FXN-deficient and depolarized control neurons. **(C)** Representative images of MitoSOX™ stained neurons (red) and β-tubulin III cytoskeleton immunostaining (green) to reveal axonal morphology were performed. We observed axonal spheroids formation (arrow) associated with ROS production in FXN-deficient, C57 H_2_O_2_ and C57 depolarized neurons. Mean ± SEM of at least three independent experiments. One way analysis of variance (ANOVA; genotype) and Bonferroni *post hoc* test were used to analyze significant changes in pixel intensity between genotypes and conditions. Significant *p*-values: ***p ≤ 0.001 were considered. 40× amplification. Scale bars, 50 μm.

To study the role of mitochondrial dysfunction in the pathological process of neuronal degeneration in FRDA, sensory neurons were isolated from control, YG8R and YG8YG8R age-matched mice (22/24-month old). Δψ_m_ and oxidative stress were analyzed in neurons under defined conditions for 5 DIV. We investigated whether frataxin deficiency effects disturb the Δψ_m_ in DRG neurons using the JC-1 ratiometric probe by confocal microscopy. The JC-1 ratio 590/530 was significantly lower in both frataxin-deficient neurons (YG8R = 1.73 ± 0.06 and YG8YG8R = 1.82 ± 0.04) compared to the control (2.19 ± 0.06), indicating decreased Δψ_m_ in these neurons (Figure 3A and Supplementary Figure S2). The C57 control neurons treated with CCCP-oligomycin, an uncoupler of mitochondrial membrane potential, exhibited the same reduction in JC-1 ratio (1.76 ± 0.04) as frataxin-deficient neurons (Figure 3A and Supplementary Figure S2) confirming mitochondrial depolarization.

Then, to clarify the role of Δψ_m_ loss in oxidative stress, we decided to measure the ROS production by loading neurons with the MitoSOX™ probe. MitoSOX™ oxidation was significantly higher in both frataxin-deficient neurons (YG8R = 213.9 ± 2.3 and YG8YG8R = 220.7 ± 2.0) compared to the control (160.4 ± 2.7). Likewise, control neurons treated with H_2_O_2_ (213.1 ± 3.0) or CCCP-oligomycin (186.9 ± 3.2) showed an increase in ROS production (Figure 3B). Thus, the increase in oxidative stress in control neurons treated with CCCP-oligomycin and frataxin-deficient neurons is associated with the loss of Δψ_m_. In addition, the higher ROS levels in frataxin-deficient neurons compared with CCCP-oligomycin control neurons suggest that the lack of frataxin causes oxidative stress, in part by decreasing the Δψ_m_ (Figure 3B).

Control neurons treated with H_2_O_2_ or CCCP-oligomycin showed axonal spheroids, which suggests that the pathological changes due to oxidative stress and mitochondrial depolarization in frataxin-deficient neurons are involved in axonal spheroid formation (Figure 3C).

### Frataxin Deficiency Involves Ca^2+^ Dyshomeostasis without Calpain Activation

**Figure 4:**
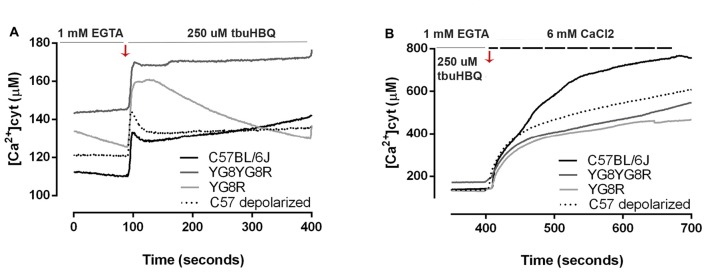
Calcium homeostasis in sensory neurons of the FRDA mouse model. Fura-2 AM fluorescence was detected in neuronal cell bodies by confocal microscopy in live cultured sensory neurons and changes in fluorescence in Ca^2+^ free medium were represented as [Ca^2+^]_cyt_ (μM). **(A)** In response to endoplasmic reticulum (ER)-Ca^2+^ depletion with 150 μM tbuHBQ, the recovery slopes showed differences in calcium buffering between genotypes. **(B)** The induction of store-operated calcium entry (SOCE) mechanism after ER-Ca^2+^ depletion was analyzed with Fura-2 [Ca^2+^]_cyt_ signals upon the re-addition of 6 mM CaCl_2_ after stimulation 6 min before with 150 μM tbuHBQ. The slope increment showed the differences in calcium entry between genotypes. Traces represent means of 32, 31, 40 and 31 total neurons measured in control, YG8YG8R, YG8R and control treated with CCCP-oligomycin (C57 depolarized), respectively, from no less than three experiments.

We investigated whether frataxin deficiency induces abnormal changes in neuronal calcium homeostasis. Neurons were loaded with FURA-2 AM and alterations in [Ca^2+^]_cyt_ were assessed in real-time in the neuronal soma. We observed that in Ca^2+^-free medium, resting cytosolic [Ca^2+^] is mildly increased in YG8R (129.5 ± 17.2 μM), YG8YG8R (144.4 ± 19.7 μM) and control neurons treated with CCCP-oligomycin (121.0 ± 27.2 μM) compared with control neurons (111.2 ± 30.5 μM; Figure 4A). Neurons were induced by the addition of t-buHBQ to the medium, a sarco-endoplasmic Ca^2+^-ATPase (SERCA) inhibitor, to obtain a complete depletion of ER Ca^2+^ stores. After [Ca^2+^]_cyt_ peaked by ER depletion, [Ca^2+^]_cyt_ recovered towards baseline in a few seconds in control neurons and CCCP-oligomycin control neurons. However, the recovery of [Ca^2+^]_cyt_ resting levels in frataxin-deficient neurons was incomplete or slower than in control (Figure 4A), possibly because of the impaired mitochondrial buffer capacity showed in frataxin deficiency (Bolinches-Amorós et al., 2014). The induction of SOCE by ER-calcium released was reduced in the frataxin-deficient neurons compared to control neurons (Figure 4B). These findings confirmed an abnormal buffering of intracellular Ca^2+^ levels and a defective SOCE mechanism in frataxin-deficient neurons, caused in part by mitochondrial depolarization. Insufficient calcium entry to neurons by SOCE after each stimulus results in inefficient ER Ca^2+^ store refilling. It could lead to inadequate neuronal calcium signaling, thereby affecting important processes such as synaptic transmission, neuronal plasticity or sensory stimulation during sense.

**Figure 5:**
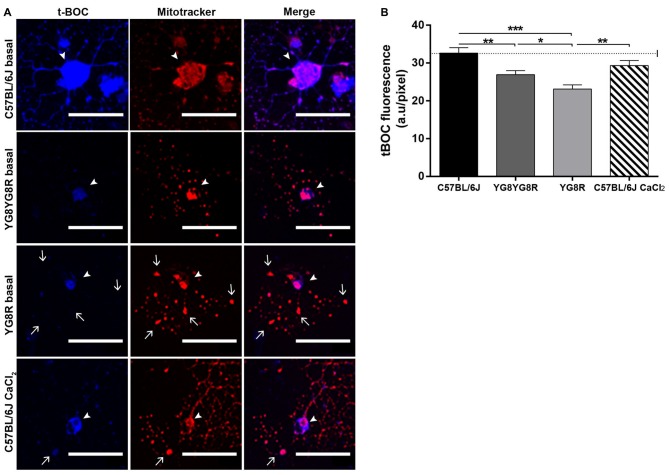
Detection of calpain activity in sensory neurons of the FRDA mouse model.** (A)** Representative images of t-BOC production dependent on calpain activity (blue) and Mitotracker stain (red) revealed mitochondrial morphology by confocal microscopy in live sensory neurons. We observed axonal spheroids formation (arrow) in FXN-deficient and Ca^2+^ overloaded control neurons. The arrowheads indicate neuronal cell bodies. **(B)** Quantitative analysis of t-BOC blue fluorescence intensity relative to neuronal soma area (pixel). Mean ± SEM of at least three independent experiments in control, YG8YG8R, YG8R and control treated with CaCl_2_ (63, 110, 106 and 73 total neurons measured respectively). We observed lower calpain activity in FXN-deficient than in control neurons. One way ANOVA (genotype) and Bonferroni *post hoc* test were used to analyze significant changes in pixel intensity between genotypes and conditions. Significant *p*-values: *p ≤ 0.05; **p ≤ 0.01; ***p ≤ 0.001. Magnification, 40× oil immersion. Scale bars, 50 μm.

Calpain is a Ca^2+^-dependent thiol protease linked mechanistically to the axonal degeneration in neurological diseases. To investigate whether elevated axoplasmic Ca^2+^ levels in frataxin-deficient neurons activate calpain and participate in cytoskeleton alterations and axonal spheroid formation, we examined calpain activity in cultured neurons at 3 DIV. Neurons were labeled with Mitotracker and the calpain substrate CMAC (CMAC, t-BOC-Leu-Met) to visualize mitochondria and calpain activity *in vivo*, respectively, by confocal microscopy. Mitotracker staining revealed a normal mitochondrial network well distributed along neurites in control neurons and a mitochondrial accumulation in axonal spheroids in frataxin-deficient neurons (Figure 5A). In basal culture conditions, the cleaved fluorescent product t-BOC in neuronal somas was more concentrated in control neurons (32.63 ± 1.43) than in both frataxin-deficient neurons (YG8R = 23.10 ± 1.11 and YG8YG8R = 26.91 ± 1.09; Figure 5B). These results indicated that calpain activity was lower in frataxin-deficient neurons than in control neurons. On the other hand, control neurons pre-treated for 24 h with CaCl_2_ showed axonal spheroids (Figure 5A) without increased calpain activity (Figure 5B). This result suggests that the increase in intra-axonal calcium levels, but not calpain activation, is involved in axonal spheroids formation in frataxin-deficient sensory neurons.

### Frataxin Deficiency Causes Alterations in Mitochondrial Network Related with Mitochondrial Depolarization and Elevated Calcium Levels

We have shown evidence that under frataxin deficiency conditions, dysfunctional mitochondria were accumulated in axonal spheroids as a marker of axonal injury. We performed an analysis of neurons to determine the effect of frataxin deficiency in mitochondrial morphology. In order to evaluate the mitochondrial morphology, we used the mito-morphology macro of ImageJ.

**Figure 6:**
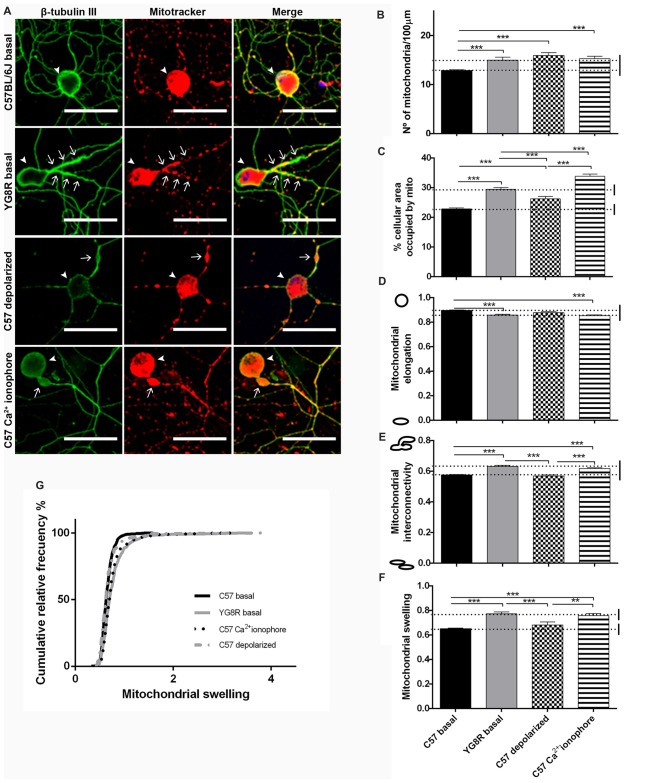
Mitochondrial and neuritic network patterns are altered in frataxin-deficient sensory neurons.** (A)** Representative images of cultured sensory neurons labeled with β-tubulin III as neuronal marker (green) and Mitotracker (red) in basal culture of frataxin-deficient and control neurons and overloaded intra-axonal calcium control. The arrows indicate axonal spheroids and arrowheads the neuronal cell bodies. Magnification, 40× oil immersion. Scale bars, 50 μm. **(B–F)** Quantitative analysis of mitochondrial parameters: **(B)** number of mitochondria; **(C)** % of cellular area occupied by mitochondria; **(D)** mitochondrial elongation; **(E)** network interconnectivity; **(F)** mitochondrial swelling; **(G)** mitochondrial swelling distribution. The first 50 μm proximal segments of axons were analyzed in control, YG8R, C57 treated with CCCP-oligomycin and C57 treated with A23 (185, 197, 59 and 99 total neurons measured respectively from at least three independent experiments). One way ANOVA (genotype) was used to analyze significant changes between genotypes and conditions. Significant *p*-values: **p ≤ 0.01; ***p ≤ 0.001.

Control neurons and frataxin-deficient neurons were grown for 5 DIV in basal culture conditions. Moreover, control neurons were treated with CCCP-oligomycin or A23 ionophore for 30 min before fixation. At 5 DIV, the mitochondria of control neurons in basal culture showed homogenous distribution in proximal and distal axonal segments (Figure 6A). In contrast, either the frataxin-deficient neurons or the control neurons treated with A23 increased the number of mitochondria and the percentage of area occupied by mitochondria in proximal segments (Figures 6B,C). Both showed lower mitochondrial elongation and higher interconnectivity of the mitochondrial network than in basal control neurons (Figures 6D,E). Also, in both cases, the mitochondrial swelling was reached until double the mitochondrial size of the control neurons in some neurons (Figures 6F,G). Then, frataxin deficiency alters mitochondrial morphology as they become shorter, more rounded and swollen than the control; moreover, mitochondria are aggregated and retained in the proximal axon. This effect was exactly reproduced by Ca^2+^-overload control. However, we found a few changes in the control neurons after treatment with CCCP-oligomycin, indicating that mitochondrial depolarization did not affect mitochondrial morphology like frataxin deficiency. Consequently, we observed similar mitochondrial morphology in intra-axonal Ca^2+^ overload and in frataxin-deficient neurons, suggesting a more relevant implication of cytosolic Ca^2+^ levels in the formation of axonal spheroids.

### Ca^2+^ Chelators and Metalloprotease Inhibitors Rescue Mitochondrial Dynamics and Axonal Dystrophy in Frataxin Deficiency

Our results suggested a relevant role of Ca^2+^ in the pathophysiology of FRDA. Consequently, we looked for pharmacological targets against Ca^2+^-mediated axonal injury. To improve Ca^2+^ handling and attenuate the consequences of Ca^2+^ overload, we modulated calcium levels with Ca^2+^ chelators.

**Figure 7:**
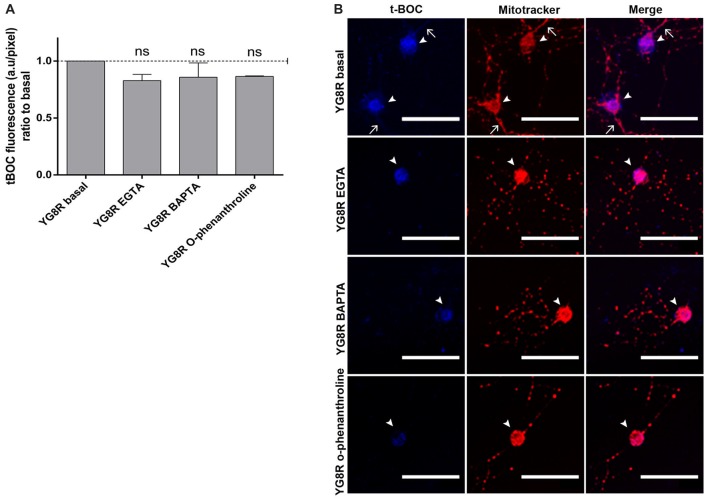
Decrease of calpain activity in sensory neurons of the FRDA mouse model under calcium chelator treatments. **(A)** Quantitative analysis of t-BOC blue fluorescence intensity relative to neuronal soma area (pixel). Mean ± SEM of at least three independent experiments in YG8R and YG8R treated with EGTA, BAPTA and *o*-phenanthroline (106, 46, 48 and 39 total neurons measured respectively). We observed lower calpain activity in YG8R treated with calcium chelators than in YG8R neurons. **(B)** Representative images of t-BOC production dependent on calpain activity (blue) and Mitotracker stain (red) to reveal mitochondrial morphology by confocal microscopy in live sensory neurons. We observed a reduction of axonal spheroids formation in calcium chelator-treated YG8R cells compared with untreated YG8R. The arrows indicate axonal spheroids and arrowheads the neuronal cell bodies. One way ANOVA (genotype) and Bonferroni *post hoc* test were used to analyze significant changes in pixel intensity between genotypes and conditions, ns: not significant. Magnification, 40× oil immersion. Scale bars, 50 μm.

YG8R neurons were grown at 2 DIV and treated for 24 h with EGTA (an extracellular Ca^2+^ chelator), BAPTA (an intracellular Ca^2+^ chelator) or *o*-phenanthroline (a chelator of metal ions). After treatments, we observed a decrease in the activity of calpain, a metal-ion-dependent enzyme, suggesting that the Ca^2+^ was chelated by BAPTA, EGTA or *o*-phenanthroline (Figure 7A). In the confocal images, the improvement in mitochondrial distribution along the YG8R neurons after the treatments can be appreciated (Figure 7B), indicating that the modulation of calcium levels could be a critical point to improve the axonopathy observed in these neurons.

**Figure 8:**
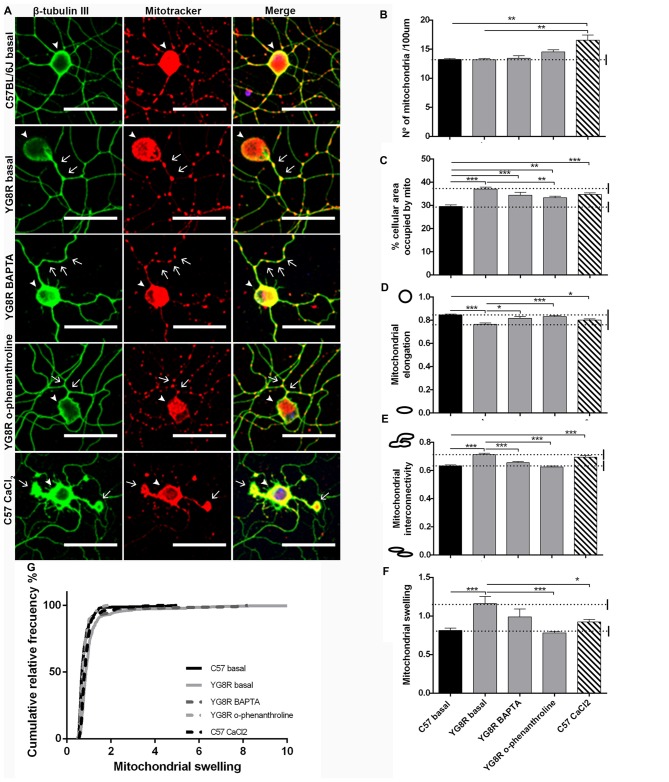
Mitochondrial and neuritic network patterns are rescued by calcium chelator treatment in sensory neurons of the FRDA mouse model.** (A)** Representative images of cultured sensory neurons labeled with β-tubulin III as neuronal marker (green) and Mitotracker (red) in basal culture of control, frataxin-deficient, YG8R treated with calcium chelators and overloaded intra-axonal calcium control. The arrows indicate axonal spheroids and arrowheads denote the neuronal cell bodies. Magnification, 40× oil immersion. Scale bar, 50 μm. **(B–F)** Quantitative analysis of mitochondrial parameters: **(B)** number of mitochondria; **(C)** % of cellular area occupied by mitochondria; **(D)** mitochondrial elongation; **(E)** network interconnectivity; **(F)** Mitochondrial swelling; **(G)** mitochondrial swelling distribution. The first 50 μm proximal segments of axons were analyzed in control, YG8R, YG8R treated with BAPTA and *o*-phenanthroline and C57 treatment with A23 (93, 99, 40, 87 and 83 total neurons measured respectively from at least three independent experiments). One way ANOVA (genotype) and Bonferroni *post hoc* test were used to analyze significant changes between genotypes and conditions. Significant *p*-values: *p ≤ 0.05; **p ≤ 0.01; ***p ≤ 0.001.

To test the effect of a reduction in calcium levels on mitochondrial morphology and axonal spheroids formation, YG8R neurons were grown with EGTA, BAPTA or *o*-phenanthroline for 4 DIV (Figure 8A). The treatment with EGTA for 4 DIV did not result in a sufficiently robust survival of YG8R neurons to allow a mitochondrial morphology study. Neuronal cultures, after immunolabeling with β-tubulin III and Mitotracker, were analyzed for mitochondrial morphology. The treatments on YG8R neurons with BAPTA and *o*-phenanthroline improved mitochondrial morphology alterations approaching control values (Figures 8B–G). Both treatments showed higher elongation and lower interconnectivity of the mitochondrial network than in YG8R basal conditions (Figures 8D,E), and decreased swelling mitochondria with values that reach sizes of the control neurons (Figures 8F,G). Computer analysis showed that control neurons treated with CaCl_2_ presented similar alterations in mitochondrial morphology than those observed in frataxin-deficient neurons (Figures 8B–G), again suggesting the relevant role of cytosolic Ca^2+^ levels in axonal damage.

## Discussion

Dying-back degeneration is common in a number of peripheral neuropathies such as FRDA or diabetic neuropathy (Höke, 2006). It is characterized by the slow progression of distal synapse loss to proximal axonal degeneration (Cavanagh, 1964) that precedes neuronal death. The nature and site of the initial injury that triggers the axonal degeneration are unknown. The dying-back neurodegeneration is present in sensory neurons of the YG8R mouse, where the origin of the pathology is in the peripheral distal nerve structures without central nervous system involvement (Mollá et al., 2016). To understand the dying-back molecular mechanism, we used the YG8R sensory neurons in culture as a model of the initial stages of degeneration.

In the *in vivo* culture model, axonal dystrophy was marked with the formation of multiple axonal spheroids and abnormal mitochondrial distribution, which support an axonal degeneration process and confirm the peripheral axonopathy showed in the YG8R mouse model (Mollá et al., 2016). Mitochondrial retention in the axonal spheroids is a hallmark of neurodegeneration and it has been described in neurodegenerative diseases in both the PNS and CNS (Coleman, 2005; Beirowski et al., 2010). In FRDA, very few studies have provided evidence of abnormalities in axons. In the *Drosophila* knock-down model, there is an early dying-back phenomenon during development related to depolarized mitochondria. The authors observed impaired retrograde axonal transport and the abnormal distribution of mitochondria in synapses (Shidara and Hollenbeck, 2010). A similar result was obtained in our model where axonal swellings in cultured YG8R sensory neurons were associated with an impairment of axonal transport, which in turn could lead to altered autophagy. Our results in frataxin-deficient sensory neurons suggest a defect in the autophagic flux more than in the activation of the process. This increase in autophagy may be associated with neurodegenerative mechanisms (Simon et al., 2004) or as a defense mechanism against oxidative stress (Bolinches-Amorós et al., 2014).

We found global mitochondrial dysfunction with a loss of mitochondrial membrane potential, oxidative stress and defective Ca^2+^ handling, which includes a failure to buffer Ca^2+^ properly and a defective SOCE mechanism. In neurons, the mitochondria can quickly buffer Ca^2+^ and then release it slowly, thus contributing to the cytosolic Ca^2+^ levels maintenance, which is extremely important in different processes such as synaptic transmission. In addition, the mitochondrial calcium uptake could be modulated by the mitochondrial membrane potential (Boitier et al., 1999). The formation of axonal spheroids may be the direct consequence of Ca^2+^ imbalance in association with ROS production and mitochondrial depolarization. A direct link between ROS production and calcium overload with axonal spheroids has also been demonstrated in other neuronal models (Nikić et al., 2011; Barsukova et al., 2012).

The Ca^2+^ modulation has been shown to be more effective than calpain inhibition (Beirowski et al., 2010; Fischer-Hayes et al., 2013) to prevent spheroids formation in neuronal models. In FRDA fibroblast and transient frataxin-deficient DRG culture, the intracellular Ca^2+^ chelation protects against oxidative stress-mediated death (Wong et al., 1999) and neurodegeneration (Mincheva-Tasheva et al., 2014). In our model, prolonged treatments for 4 DIV with BAPTA and *o*-phenanthroline reverted the axonal dystrophy and morphology of the mitochondrial network. These results support the important idea that the axonal pathology is reversible in FRDA by the intracellular Ca^2+^ handle, preventing Ca^2+^-mediated axonal injury. The metalloproteases inhibitor *o*-phenanthroline is more effective in reverting axonal dystrophy than the intracellular Ca^2+^ chelator BAPTA, despite a similar inhibition of calpain activity. Thus, calpain-independent mechanisms are improving axonal dystrophy, possibly by inhibiting other metalloproteases (Ma, 2013) or by chelating iron overload related with frataxin deficiency. In fact, the protection of axons in chronic neurodegenerative disease by calpain inhibition remains to be demonstrated (Fischer-Hayes et al., 2013).

In the course of FRDA, axonal damage precedes neuronal cell body death and it seems the most likely cause of dysfunction, suggesting the occurrence of dying-back processes. Until now, however, the way in which damage is initiated and propagated remains unclear. Our results in YG8R sensory neurons have given us novel clues about the mechanism of axonal multifocal degeneration, where altered Ca^2+^ handling is critical in the initial stages of dying-back neurodegeneration. An effective development of safety treatments to prevent Ca^2+^-mediated axonal injury is a challenge to be studied in FRDA.

## Author Contributions

BM conducted and designed experiments, analyzed the results and wrote the manuscript. FR performed the experiments. AB-A and DCM-L contributed to performing the experiments and interpreting the data. MI-V and AF-V customized mito-morphology macro of ImageJ for morphometric mitochondrial analysis. FVP interpreted the data and wrote the manuscript. FP and PG-C designed the study, supervised the experiments, analyzed the data and wrote the manuscript. All authors read and approved the final manuscript.

## Conflict of Interest Statement

The authors declare that the research was conducted in the absence of any commercial or financial relationships that could be construed as a potential conflict of interest.

## Supplementary Material

FIGURE S1 Detection of human frataxin levels in neuronal tissue of FRDA mouse model. Representative western blot of human FXN expression in all genotypes of FRDA mouse model. Human FXN was only detected in YG8R and YG8YG8R mice, in which the transgene was present. The FXN level was higher in the YG8YG8R mouse because has double of the transgene than YG8R.

FIGURE S2 Detection of Δψ_m_ in sensory neurons of the FRDA mouse model. Representative images of JC-1 stained neurons (green) detected by confocal microscopy in cultured sensory neurons of control, YG8YG8R, YG8R and control treated with CCCP-oligomycin (C57 depolarized; 182, 182, 129 and 87 total neurons measured respectively from no less than three experiments) are shown. We observed mitochondrial depolarization in FXN-deficient and depolarized control neurons. The arrows [↑] indicate axonal spheroids and the arrowheads indicate neuronal cell bodies, 40× amplification, Scale bars, 10 μm. Quantitative analysis of JC-1 fluorescence intensity is represented in Figure 3A. Some images are saturated in order to clearly show changes in the mitocondrial potential in axons and spheroids.
