# Supplementary figures and images for: Reversible Axonal Dystrophy by Calcium Modulation in Frataxin-Deficient Sensory Neurons of YG8R Mice
Source: Front Mol Neurosci. 2017 Aug 30;10:264. doi: 10.3389/fnmol.2017.00264 (PMC5583981; doi:10.3389/fnmol.2017.00264)

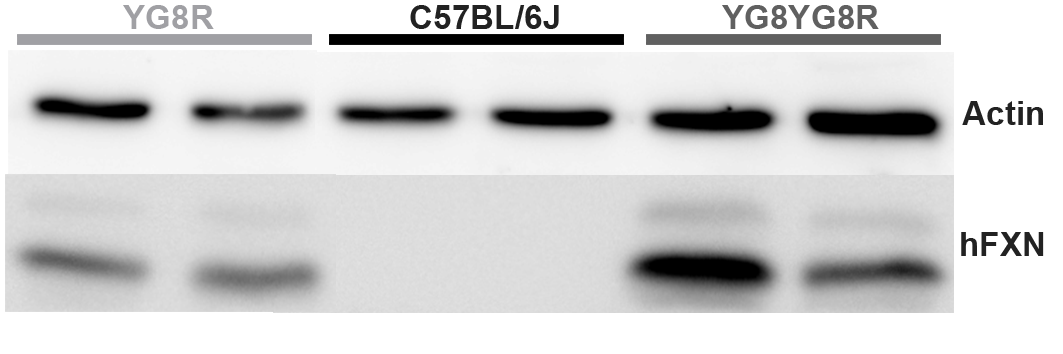

Supplement: FIGURE S1 — Detection of human frataxin levels in neuronal tissue of FRDA mouse model. Representative western blot of human FXN expression in all genotypes of FRDA mouse model. Human FXN was only detected in YG8R and YG8YG8R mice, in which the transgene was present. The FXN level was higher in the YG8YG8R mouse because has double of the transgene than YG8R. [file Image_1.tif]

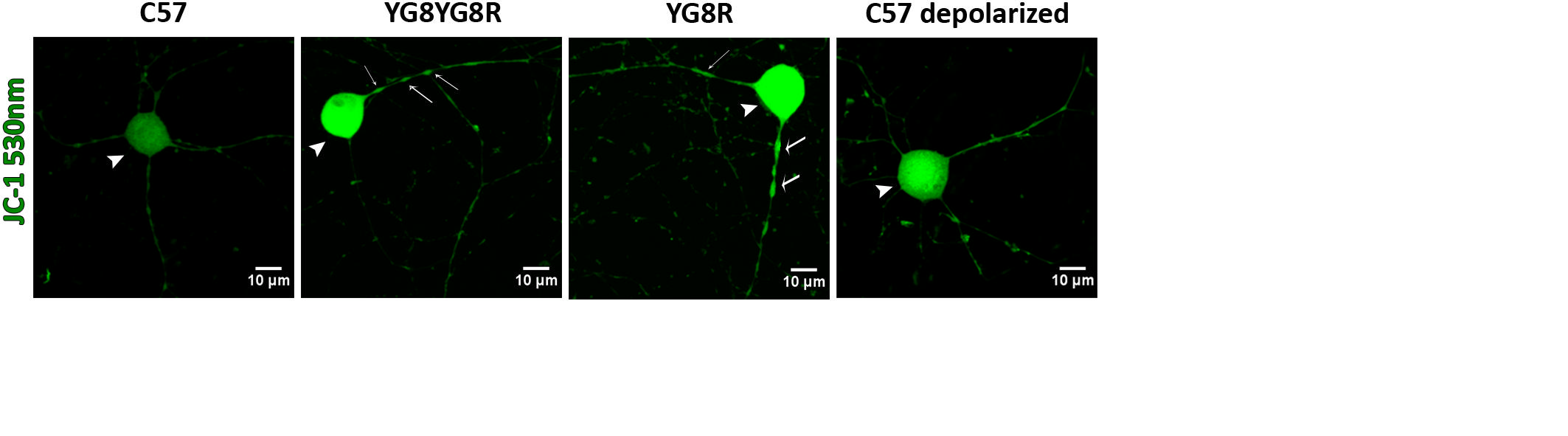

Supplement: FIGURE S2 — Detection of Δψm in sensory neurons of the FRDA mouse model. Representative images of JC-1 stained neurons (green) detected by confocal microscopy in cultured sensory neurons of control, YG8YG8R, YG8R and control treated with CCCP-oligomycin (C57 depolarized; 182, 182, 129 and 87 total neurons measured respectively from no less than three experiments) are shown. We observed mitochondrial depolarization in FXN-deficient and depolarized control neurons. The arrows [↑] indicate axonal spheroids and the arrowheads indicate neuronal cell bodies, 40× amplification, Scale bars, 10 μm. Quantitative analysis of JC-1 fluorescence intensity is represented in Figure 3A. Some images are saturated in order to clearly show changes in the mitocondrial potential in axons and spheroids. [file Image_2.tif]
